# The Landscape of Repetitive Elements in the Refined Genome of Chilli Anthracnose Fungus *Colletotrichum truncatum*

**DOI:** 10.3389/fmicb.2018.02367

**Published:** 2018-10-04

**Authors:** Soumya Rao, Saphy Sharda, Vineesha Oddi, Madhusudan R. Nandineni

**Affiliations:** ^1^Laboratory of Genomics and Profiling Applications, Centre for DNA Fingerprinting and Diagnostics, Hyderabad, India; ^2^Graduate Studies, Manipal Academy of Higher Education, Manipal, India; ^3^Laboratory of Cell Signalling, Centre for DNA Fingerprinting and Diagnostics, Hyderabad, India; ^4^Laboratory of DNA Fingerprinting Services, Centre for DNA Fingerprinting and Diagnostics, Hyderabad, India

**Keywords:** *Colletotrichum truncatum*, whole genome sequence, repetitive DNA sequences, transposable elements (TEs), simple sequence repeats (SSRs), comparative genomics

## Abstract

The ascomycete fungus *Colletotrichum truncatum* is a major phytopathogen with a broad host range which causes anthracnose disease of chilli. The genome sequencing of this fungus led to the discovery of functional categories of genes that may play important roles in fungal pathogenicity. However, the presence of gaps in *C. truncatum* draft assembly prevented the accurate prediction of repetitive elements, which are the key players to determine the genome architecture and drive evolution and host adaptation. We re-sequenced its genome using single-molecule real-time (SMRT) sequencing technology to obtain a refined assembly with lesser and smaller gaps and ambiguities. This enabled us to study its genome architecture by characterising the repetitive sequences like transposable elements (TEs) and simple sequence repeats (SSRs), which constituted 4.9 and 0.38% of the assembled genome, respectively. The comparative analysis among different *Colletotrichum* species revealed the extensive repeat rich regions, dominated by Gypsy superfamily of long terminal repeats (LTRs), and the differential composition of SSRs in their genomes. Our study revealed a recent burst of LTR amplification in *C. truncatum*, *C. higginsianum*, and *C. scovillei*. TEs in *C. truncatum* were significantly associated with secretome, effectors and genes in secondary metabolism clusters. Some of the TE families in *C. truncatum* showed cytosine to thymine transitions indicative of repeat-induced point mutation (RIP). *C. orbiculare* and *C. graminicola* showed strong signatures of RIP across their genomes and “two-speed” genomes with extensive AT-rich and gene-sparse regions. Comparative genomic analyses of *Colletotrichum* species provided an insight into the species-specific SSR profiles. The SSRs in the coding and non-coding regions of the genome revealed the composition of trinucleotide repeat motifs in exons with potential to alter the translated protein structure through amino acid repeats. This is the first genome-wide study of TEs and SSRs in *C. truncatum* and their comparative analysis with six other *Colletotrichum* species, which would serve as a useful resource for future research to get insights into the potential role of TEs in genome expansion and evolution of *Colletotrichum* fungi and for development of SSR-based molecular markers for population genomic studies.

## Introduction

*Colletotrichum truncatum* (syn. *C. capsici*), belonging to one of the most common and important genera of phytopathogenic ascomycete fungi, causes fruit rot or anthracnose disease in chilli (Dean et al., 2012), which is a major concern for many chilli-producing countries of the world, including India (Than et al., 2008). It has a wide host range with more than 400 hosts including many economically important crops from the families *Amaranthaceae, Asteraceae, Brassicaceae, Cyperaceae, Euphorbiaceae, Fabaceae, Malvaceae, Oleaceae, Poaceae, Rosaceae, Solanaceae* etc. (Jayawardena et al., 2016). The *Colletotrichum* species usually exhibit hemibiotrophic lifestyle to infect the host plants, whereas *C. truncatum* adopts subcuticular intramural necrotrophic lifestyle during interaction with chilli (with short endophytic phase) and other hosts like cotton, cowpea etc. (Perfect et al., 1999; Ranathunge et al., 2016). A pathogen with such a broad host range and varied lifestyle might have evolved constantly and adapted to different niches over the time in a continuous armed race with different hosts. In order to establish successful interactions with a variety of hosts, pathogenic fungi employ diverse mechanisms to cross physical and chemical barriers of host immune system and secrete effector proteins to counteract defence mechanisms by manipulating the host gene expression (Lo Presti et al., 2015). There is a constant need for the pathogen to evolve novel effectors and modify the existing ones to avoid the host recognition.

Fungal pathogens have highly dynamic genomes that show high variability in size and composition even within closely-related species, primarily due to genomic rearrangements and differences in repetitive DNA content (Seidl and Thomma, 2014; Möller and Stukenbrock, 2017). The genome expansion and plasticity in eukaryotic organisms is typically driven by the activity of repetitive elements like transposons, deletion, translocation, duplication of genomic content, recombination in sexually propagating organisms, etc. (Seidl and Thomma, 2014). The genome plasticity and repetitive elements in the genomes represent the evolutionary forces in play and the capacity to adapt to dynamic environmental conditions (Wostemeyer and Kreibich, 2002). The most important category of repetitive elements is represented by transposable elements (TEs), the ubiquitous and mobile genetic elements which are capable of self-replication and propagation within a genome (Bowen and Jordan, 2002). Once considered as “junk DNA,” the activity of these elements has been shown to have major consequences on genome organisation, function, and evolution of eukaryotes (Wessler, 2006; Seidl and Thomma, 2017).

Transposable elements are broadly divided into two classes based on their structural features and mode of transposition: Class I TEs or retrotransposons, generally function via reverse transcription and propagate through copy-and-paste mechanism; while Class II TEs or DNA transposons use transposase enzyme activity to propagate through cut-and-paste mechanism (Wicker et al., 2007). Class I elements are further classified into long terminal repeats (LTRs) and non-LTR elements; while Class II elements are classified mainly into a subclass containing terminal inverted repeats (TIRs) and Cryptons, and the other including Helitrons and Mavericks (Wicker et al., 2007). LTRs, especially the Gypsy and Copia superfamilies, are the most abundant, ubiquitous and widely studied TEs that are the key drivers of genome size expansion in eukaryotes, doubling their copy numbers at each transposition event (Elliott and Gregory, 2015). Although there have been comprehensive studies on DNA elements also, these elements form a minor fraction of repeats in the fungal genomes (Muszewska et al., 2017), mainly represented by Tc1/Mariner, hAT and Helitron elements (Elliott and Gregory, 2015).

Changes in the content and composition of transposable elements bring about considerable changes in the genome architecture. The host adaptability and chromosomal rearrangements were found to be associated with the diversity in the pathogenic populations arising due to different factors including transposition and recombination. TEs were proposed to affect the host genomes by modulating their size and regulating gene expression and function and giving rise to novel genes (Möller and Stukenbrock, 2017). Because of the major role of TEs in shaping the genome structure and gene regulation, their identification and characterisation provides a wealth of useful data to gain an in-depth understanding of genome structure and function. Most of the fungi have 1–25% of repetitive DNA content (Castanera et al., 2016) and their genome size is positively correlated with the number of transposon families hosted within the genome (Elliott and Gregory, 2015). The TE silencing mechanisms like DNA methylation (Goll and Bestor, 2005; Zemach et al., 2010), repeat induced point mutation (RIP) (Cambareri et al., 1989) and RNA interference (RNAi) (Fulci and Macino, 2007) limit the excessive TE activity, which could be harmful to the organism in case the housekeeping genes are mutated due to transposition. DNA methylation has been reported to be an active epigenetic mechanism to control the TE proliferation in fungi and other eukaryotes (Zemach et al., 2010). There are several fungi in which RIP induces C to T mutations in the TEs and often in their neighbouring genes as well, thus affecting their activity and expression. It was proposed that RIP gives rise to the AT-rich, gene-poor repeat islands and promote the “two-speed” genomes in several fungi (Cambareri et al., 1989; Santana et al., 2012, 2014; Dhillon et al., 2014; Li et al., 2017). RNAi mediated gene silencing or quelling is generally observed in sexual species during meiosis if an unpaired TE is present in either of the parental chromosomes.

In addition to TEs, simple sequence repeats (SSRs) or microsatellites, typically composed of 1–6 nucleotide long repetitive units, are the other major type of the repetitive elements ubiquitous in all organisms (Tóth et al., 2000; Karaoglu et al., 2005). SSRs show a high variability in the number of repeats due to insertion or deletion of repeat motifs during DNA replication. They also play a vital role in gene regulation, chromatin organisation and contribute to genome evolution by creating genetic variations (Cavagnaro et al., 2010). Owing to their multiallelic nature, SSRs display a high degree of polymorphism, especially for long repetitive loci, and are therefore widely used as molecular markers for the population genomic studies, DNA fingerprinting and diversity studies in both prokaryotes and eukaryotes (Lim et al., 2004). Some molecular marker technologies like RAPD, RFLP, AFLP, and ISSRs were conventionally used in fungi and plants but these methods are often laborious, time consuming, cost intensive and poorly reproducible due to anonymous DNA fragments that may vary in sequence despite similar sizes (Agarwal et al., 2008). On the other hand, the codominant SSR markers have high reproducibility and transferability among related species, and are more informative than other markers due to their multi-allelic nature (Vieira et al., 2016).

The availability of whole genome sequences of many organisms has enabled the researchers from all over the world to use these genomic resources for a variety of purposes. It also led to the development of high throughput *in silico* methods to identify SSRs from the whole genome sequences. SSR discovery in the fungal genomes has helped in the evaluation and analysis of inter- and intra-species variations, development of novel markers for characterisation of different fungal populations and evolutionary and functional studies. However, there have been only limited studies on composition of SSRs in the sequenced genomes of fungi like yeast, mushrooms and some phytopathogenic fungi including *Fusarium, Magnaporthe* species, *Aspergillus* species etc. (Lim et al., 2004; Wang et al., 2014; Qu et al., 2016; Mahfooz et al., 2017). The SSR profiles of different fungal species and strains studied so far were found to be highly specific in terms of the SSR types, frequency of occurrence, density and types of dominant motifs, independent of their genome sizes (Karaoglu et al., 2005).

With the advent of cheaper and faster next generation sequencing technologies, whole genome sequences of several phytopathogenic fungi are publically available now and have provided a glimpse into their genome architecture, repeat structure and composition. However, only a handful of fungal phytopathogens have their finished or nearly complete genome sequences available publically. Hence, the missing information on repetitive regions hinders the in-depth study of genome structure, evolution and speciation of these organisms. The genome sequences of many *Colletotrichum* species are available in public databases but most of these are fragmented into several contigs or scaffolds. Although a few species have relatively less fragmented genomes, like *C. scovillei* (Han et al., 2016) and *C. truncatum* (Rao and Nandineni, 2017) with 34 and 80 scaffolds, respectively. Only *C. higginsianum* is among a few phytopathogenic fungi for which nearly-complete genome sequence is publically available (11 complete chromosomes, 1 incomplete chromosome and 12 unitigs) (Zampounis et al., 2016; Dallery et al., 2017).

A high quality genome sequence of *C. truncatum* is available which provides a glimpse of the genome composition and functionally important categories of genes (Rao and Nandineni, 2017). However, there were several gaps in the existing draft assembly which was sequenced on an Illumina platform. The missing sequences could be attributed to the repetitive elements that are difficult to assemble. The emergence of long read sequencing technologies such as Pacific Biosciences (PacBio) single-molecule real-time (SMRT) sequencing and Oxford Nanopore sequencing have greatly enabled completion of genome assemblies by generating long reads upto 60 kilobases (kb), which often span repetitive elements (Rhoads and Au, 2015). Thus, long read sequencing methods help in filling the gaps and completion of the genome assemblies and to ascertain sequence accuracy, either by using hybrid approaches or high-resolution optical mapping, to achieve telomere-to-telomere assembly of different chromosomes (Dallery et al., 2017).

The completion of genome assemblies and annotation is essential to study the important features of evolutionary and functional genomics of individual species and genera, including their repetitive element landscape. We carried out SMRT sequencing with long reads to supplement the draft assembly in order to fill the gaps and get a more refined genome sequence of *C. truncatum*. This provided an opportunity to study the repetitive element landscape and genome architecture in *C. truncatum* and to compare it with six other *Colletotrichum* species with relatively less fragmented genomes. This study provides a glimpse into the composition and distribution of TEs and SSRs in this fungal genus and a resource for future evolutionary and functional studies, manual curation of repeat families and development of molecular markers.

## Materials and Methods

### PacBio Sequencing and Assembly of *C. truncatum* Genome

*Colletotrichum truncatum* culture, originally isolated from chilli in Puducherry, India, was procured from the Microbial Type Culture Collection (MTCC), Institute of Microbial Technology, Chandigarh, India (MTCC no. 3414). The genomic DNA was isolated from *C. truncatum* culture grown for 3 days at 28°C in potato dextrose broth (PDB) using DNeasy Plant Minikit (Qiagen, Hilden, Germany) according to the manufacturer’s instructions. The DNA was sheared using G-tube (Covaris, Inc., Woburn, MA, United States) to ∼10 kb of insert size. SMRTbell template library was prepared according to PacBio protocols and sequenced using the PacBio RSII instrument (Pacific Biosciences, Menlo Park, CA, United States) at the Max Planck Institute for Evolutionary Anthropology, Leipzig, Germany. The library was sequenced on three single-molecule real-time (SMRT) cells using the P6-C4 polymerase-chemistry at 50, 65, and 75 pM DNA concentrations. The hybrid scaffolding approach was used to map the filtered subreads, obtained with AHA module of SMRT portal of PacBio, on the Illumina assembly of *C. truncatum* (Rao and Nandineni, 2017) and to fill the gaps within and between the scaffolds using PBJelly version 14.9.9^1^ with default parameters. The refined assembly obtained with PBjelly was used for the subsequent analyses. Completeness of the assembly was evaluated through BUSCO (Simão et al., 2015) by using conserved Sordariomycete gene sets.

### Gene Annotation

MAKER2 pipeline was used for gene prediction from the refined genome (Campbell et al., 2014). It was based on the evidence from RNA-Seq data obtained from three *in vitro* [*C. truncatum* cultures grown on potato dextrose agar (PDA), Czapeck’s medium (CZ) and appresorial assay (APR)] and two *in planta* samples of chilli inoculated with *C. truncatum* at 24 and 72 hour post inoculation (hpi) as described in Rao and Nandineni (2017). The cleaned up paired-end RNA-Seq reads, super reads and singletons obtained from MaSuRCA using all the *in vitro* samples and those from *in planta* samples that did not map to chilli genome were mapped to the *C. truncatum* genome through HiSAT2 (Kim et al., 2015) and assembled into transcripts through StringTie version 1.2.3 (Pertea et al., 2015). The homologs from other *Colletotrichum* species and *ab initio* gene predictions by AUGUSTUS version 3.0.3 (Stanke et al., 2006), trained on the BUSCO output, and SNAP version 2013-02-16 (Korf, 2004) were also used to get consensus gene models. The secretome prediction was carried out using the same pipeline of tools (SignalP 4, Phobias, WoLF PSORT, PredGPI and PSscan) as described previously (Rao and Nandineni, 2017). EffectorP was used to predict putative effectors in the secretome (Sperschneider et al., 2016). SMURF online tool was used to identify secondary metabolite gene clusters (Khaldi et al., 2010).

### Identification and Characterisation of Repetitive Elements

A custom library of *de novo* repeats was generated from the refined genome assembly using RepeatModeler version 1.0.8^2^. The identified repeat families were screened for protein coding genes using ProtExcluder version 1.1 to remove the sequences that have homologs in the UniProtKB/SwissProt database. The resulting *de novo* repeat library, consisting of 48 repetitive elements was combined with repeat peptide database, Repbase (update 22.9.2017) to be used as final library consisting of 50,847 repeats. RepeatMasker version 4.0.5^3^ was run on sensitive mode with rmblastn version 2.2.27+, to identify families of repetitive sequences in the *C. truncatum* genome based on the homology with the repeats in the final library. Both complete and incomplete repeat elements were detected by RepeatMasker. The unspecified elements that had only partial matches were classified based on their homologies to repeat peptides in Repbase. The summary statistics of the repetitive elements were obtained using buildSummary.pl script available in RepeatMasker utilities.

In order to estimate the age of LTR insertion, the full-length LTRs retrotransposons were identified through LTRharvest (Ellinghaus et al., 2008). 5′ and 3′ LTRs from each of the LTR element were aligned using ClustalW and Kimura 2-parameter distance (*k*) was calculated for each pair using dnadist programme of PHYLIP (Retief, 2000). The divergence time (*T*) was calculated using the formula *T* = *d*/2*r*, where *r* is the fungal substitution rate of 1.05 × 10^-9^ nucleotides per site per year (Castanera et al., 2016).

OcculterCut version 1.1 was used to detect the GC-bias in the genome with default setting (Testa et al., 2016). RIPCAL version 2 was used to calculate the dinucleotide frequency across the genome and the two RIP indices, TpA/ApT and (CpA + TpG)/(ApC + GpT) (Hane and Oliver, 2008). The RIP indices were also calculated for the most prevalent TE families with at least 5 copies >400 bp in length and >80% identity to the longest element taken as reference sequence in all vs. all BLASTn analysis. The sequences in each family were aligned and manually edited through MEGA 7.0.9 (Kumar et al., 2016) and were subjected to RIPCAL analysis.

To analyse the association between repetitive elements and secreted proteins, putative effectors and all the genes in SM clusters, their distances from nearest repeats were determined using the “closest” module of BEDTools (Quinlan and Hall, 2010). The distances generated for the effectors, genes in secretome and SM gene clusters were compared to the distances for sets of random genes with similar sizes as each gene category using Wilcoxon’s test in R version 3.3.0^4^. A permutation test implemented in the R package RegioneR (Gel et al., 2015) was used to compare the mean distances between TEs and the above three gene categories with mean distance of a random sample of genes generated from whole genome with 10,000 permutations. A set of 1,000 random genes was used as negative control for the test.

Simple sequence repeat identification was accomplished using MIcroSAtellite identification tool (MISA)^5^ with default parameters: mononucleotide repeat motif with at least ten repeats, dinucleotide motif with six repeats, tri-, tetra-, penta-, and hexa-nucleotide motifs with five repeats. Compound microsatellites had two repeat motifs within 100 bases. *Ascochyta rabiei* was taken as a reference for ascertaining the accuracy of SSR detection by MISA (Verma et al., 2016). The sequences of exons, introns and intergenic regions were obtained from the .gff file using the Bedtools. MISA was run separately for each category of genomic regions separately. The relative abundance for each SSR type was calculated as the number of repeats per Mb of genome, while density was calculated as the number of bases for each SSR type per Mb of genome. The analysis of repeat motifs of each SSR type that occurred more frequently was carried out by treating circular permutations of unit pattern as equivalent.

### Comparative Genomic Analyses

The identification and comparative analyses of repeat families, TEs, SSRs, RIP indices and GC-bias were performed with the nearly finished genomes of *C. higginsianum* and *C. scovillei*, and four other relatively less-fragmented genomes of *Colletotrichum* species (**Table 1**) using the same tools as described above.

**Table 1:** The *Colletotrichum* species used for the comparative analysis of TEs.

Organism	Host	Country of isolation	Genome size (Mb)	GC%	Number of scaffolds	Availability of gene annotation (.gff) file	Accession number (NCBI/dryad)
*C. truncatum*	*Capsicum annuum*	India	57.91	49.38	70	Yes	NBAU02000000
*C. graminicola*	*Zea mays*	United States	50.91	49.1	654	Yes	ACOD00000000.1
*C. higginsianum*	*Brassica rapa*	Trinidad and Tobago	50.72	54.4	25	Yes	LTAN00000000.1
*C. scovillei*	*Capsicum annuum*	South Korea	52.13	51.7	34	No	LUXP01000000.1
*C. chlorophyti*	*Solanum lycopersicum*	Japan	52.33	50.06	512	Yes	MPGH00000000.1
*C. orchidophilum*	*Phalaenopsis* sp.	United Kingdom	48.56	51.1	321	Yes	MJBS00000000.1
*C. orbiculare*	*Cucumis sativus*	Japan	90.83	37.52	526	No	dryad.45076


## Results

### Refined Genome Assembly and Annotation of *C. truncatum*

In order to get a refined genome assembly, long SMRT reads were used for hybrid scaffolding of the existing high quality draft genome assembly. Approximately, 2.1 Gb of raw sequence data were generated from the 166,719 polymerase reads with average read length of 12.65 kb and average insert read size of 4,616 bp. 5,26,000 PacBio filtered subreads were used to get 42× coverage. The hybrid scaffolding of the Illumina assembly with these subreads resulted in the refined genome consisting of 70 scaffolds with total size of 57,912,832 bp, showing an improvement of ∼2.3 Mb of sequence content over the previous assembly (Rao and Nandineni, 2017). The refined sequence had only 2.26% of gaps as opposed to 4.75% in the draft sequence (**Table 2**). The BUSCO analysis showed marginal improvement in the number of conserved Sordariomycete genes detected in the refined assembly as compared to the draft assembly.

**Table 2:** Summary of *C. truncatum* assemblies.

Statistics	Illumina^∗^	Illumina + PacBio^#^
Number of scaffolds	80	70
Total length (Mb)	55.37	57.91
Mean scaffold length (kb)	683.5	827
Number of gaps	6,793	3,738
Total gap length (Mb)	2.6	1.3
Mean gap length	387	351
Percent *N*’s	4.75%	2.26%
Protein coding genes	13,724	13,768
Secretome	1245	1213
Effectors	310	311
SM clusters	73	64
**BUSCO coverage**
Complete genes	3563	3576
Fragmented genes	150	141
Missing genes	12	8


*^∗^Rao and Nandineni (2017).****^#^****This study*.

13,768 protein coding genes were predicted in the refined *C. truncatum* genome, only 44 more than the previous draft version. There were 13,485 common gene models between the two versions and 11,436 genes had >99% identity, while 13,264 genes had >70% identity. Slight differences between the sizes of secretome and effector components were observed, while the number of secondary metabolite gene clusters were little less than those detected from the draft assembly (**Table 2**).

### Analysis of Transposable Elements

**FIGURE 1:**
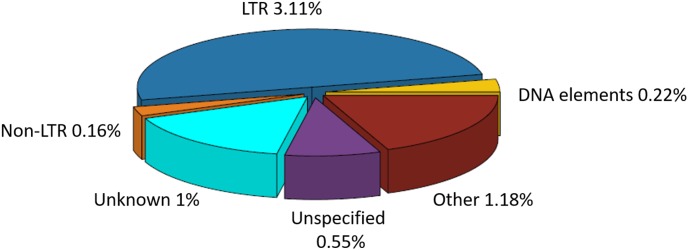
The percentage of repetitive elements and TE families in the total repeat component of *Colletotrichum truncatum* genome identified by the RepeatMasker software. LTRs occupied the largest fraction of TEs, while non-LTR and DNA elements formed the smallest. Other repetitive elements included SSRs, satellites, rDNA repeats etc.

The total repeat content in *C. truncatum* refined assembly was 6.08%, majority of which was contributed by TEs (4.89%). Almost all the repeats were ancestral repeats which could be classified at higher taxa than the species. Only a minor fraction of repeats (0.13%) were classified as lineage specific repeats that were unique to *C. truncatum*. Both Class I (LTR, non-LTR) and Class II (DNA) elements were represented in the genome. Both complete and incomplete, truncated or disrupted elements were identified, most of which were either unknown, consisting of consensus sequences with no similarity to any Repbase entry, or unspecified or incomplete, containing some but not all features of TEs (**Figure 1** and **Table 3**). Unspecified TEs could be classified based on their homology to repeat peptides in the Repbase (**Supplementary Table S1**). Among the classified TEs, Gypsy and Copia LTRs were the most abundant elements representing 3.02% of the genome, followed by DNA elements and LINEs (**Table 3**).

**Table 3:** The composition of major families of TEs in *C. truncatum.*

Class		Count	Size (bp)	Proportion of genome (%)
Total sequences		70	57912832	
Ancestral repeats		19879	3446297	5.95
Lineage specific repeats		259	74689	0.13
Total repeats		20138	3520986	6.08
Total TEs		6035	2831668	4.89
LTR	Gypsy	833	1350797	2.33
	Copia	195	397087	0.69
LINE	CRE-Cnl1	56	60017	0.10
	CRE	30	22296	0.04
SINE		120	10372	0.02
DNA	MULE-MuDR	52	93987	0.16
	TcMar-Fot1	27	21534	0.04
	PiggyBac	70	13388	0.02
Unknown		1804	559668	0.97
Unspecified		2848	302522	0.52


The comparative genomic analysis of seven *Colletotrichum* species showed that the total TE content varied widely among them, ranging from 4.3–44.8% of the genome in *C. scovillei* to *C. orbiculare*, corresponding to their genomic sizes of ∼52–91 Mb, respectively (**Table 4**). The total TE content of *C. orbiculare* was five-fold more than the TE fraction reported earlier (8.3%) (Gan et al., 2013). The unknown elements contributed the most (26%) to its high repeat content. The diverse TE landscape of all the species had only one common LTR element superfamily of Gypsy elements that formed a major TE fraction in all fungi. In *C. orbiculare*, Copia elements were the most abundant LTRs that occupied 12% of the genome, while these elements were negligible in *C. scovillei* and *C. orchidophilum*. Among the DNA elements, TcMar-Fot1 was found in all the species except *C. orbiculare*. The number and fraction of TEs in different species corresponded to their genome sizes in general.

**Table 4:** The comparison of major TE families among *Colletotrichum* species.

TE family	*C. truncatum*	*C. higginsianum*	*C. graminicola*	*C. orbiculare*	*C. scovillei*	*C. chlorophyti*	*C. orchidophilum*
Gypsy	2.33	1.23	5.18	3.94	1.51	6.12	3.41
Copia	0.69	0.68	3.16	12.08	-	2.38	-
TcMar-Fot1	0.04	1.63	2.17	-	0.65	0.26	0.48
Unknown	0.97	1.03	3.56	26.41	1.69	0.15	0.26
Unspecified	0.52	0.46	0.38	0.19	0.41	0.43	0.40
Total TEs	4.89	6.01	14.79	44.88	4.31	9.54	5.41


### Estimation of Age of Insertion of LTRs

**FIGURE 2:**
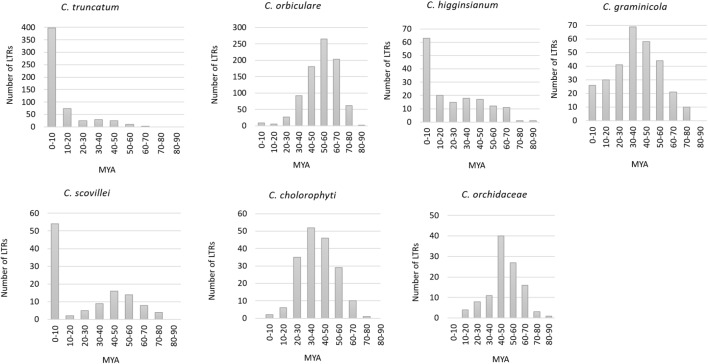
The estimated time of insertion of LTRs in the genomes of *Colletotrichum* species. It was calculated based on the sequence divergence between 5′ and 3′ LTRs of the complete elements. MYA, million years ago.

Five hundred and sixty-two complete LTR retrotransposons (putative autonomous elements) with intact LTRs were identified in *C. truncatum* genome through LTRharvest. The number of intact LTRs varied among the other species viz., *C. graminicola* (299), *C. chlorophyti* (181), *C. higginsianum* (158), *C. scovillei* (112), and *C. orchidaceae* (110). *C. orbiculare* had the highest number of LTRs (845) among the seven *Colletotrichum* species. Three of the species showed recent burst of LTR amplification in their genomes with 58.5% of the LTRs inserted in 0–3 million years ago (MYA) in *C. truncatum*, and more than 40% LTRs amplified in last 10 MY in *C. higginsianum* and *C. scovillei* (**Figure 2**). In *C. higginsianum* and *C. truncatum*, the LTR insertion rate was uniform with progressive decay from 10–70 MYA. In *C. scovillei*, a stagnant phase appeared from 10–20 MYA, wherein the lowest amplification activity was observed. In the other four species, relatively ancient bursts of LTR amplifications were observed peaking from 30–40 MYA in *C. graminicola* and *C. chlorophyti*, 40–50 MYA in *C. orchidaceae* and 50–60 MYA in *C. orbiculare*, with uniform rates of insertion in all (**Figure 2**). The oldest insertion event was estimated to have occurred at 63.83 MYA in *C. truncatum* that went upto 85.5 MYA in *C. higginsianum* and 87.5 MYA in *C. orbiculare*.

### Association of TEs With Genes

The secreted proteins, effectors and genes in secondary metabolite clusters were compared to random sets of genes of similar sizes with respect to their distance from nearest repetitive elements using Wilcoxon’s test. No significant difference was observed between the distances of these three gene categories from repetitive elements when compared to the random genes in *C. truncatum* (*p*-values = 0.18, 0.49, and 0.78 for secreted proteins, effectors and genes in secondary metabolite clusters, respectively).

**FIGURE 3:**
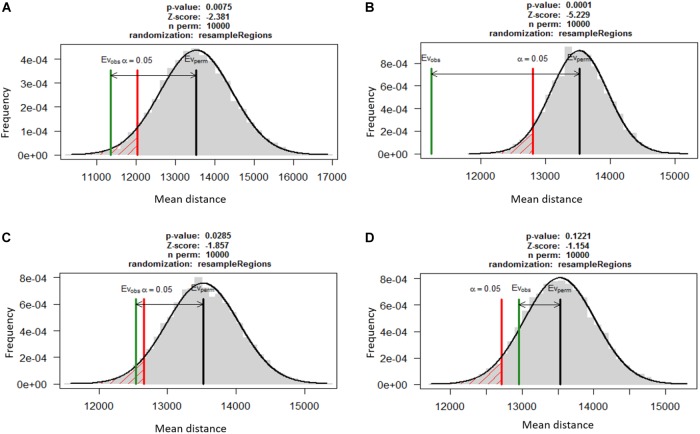
The association between TEs and three functionally relevant gene categories based on permutation test using RegioneR R package. Putative effectors **(A)**, secreted proteins **(B)** and genes in secondary metabolite clusters **(C)** were significantly closer to TEs as compared to the random genes in the genome. A set of 1,000 other genes **(D)** was taken as negative control for the test that showed these genes were not significantly associated with TEs.

A permutation test using RegioneR sampled 10,000 random permutations of genes from whole genome and compared the mean distances of TEs from random gene set to the mean distances from secretome, effectors and secondary metabolites. The distribution of means was used to calculate the p-values that showed the three gene categories were significantly more closely associated with TEs as compared to the random genes (**Figure 3**). The secreted proteins were most significantly associated to TEs (mean distance of 11,234 bp; *p* = 0.0001) as compared to the effectors (mean distance 11,356 bp; *p* < 0.001) and secondary metabolite clusters (mean distance 12,543 bp; *p* < 0.05). The random gene set showed no significant association to TEs (mean distance 12,956 bp; *p* > 0.1).

### Detection of GC-Bias and RIP

**FIGURE 4:**
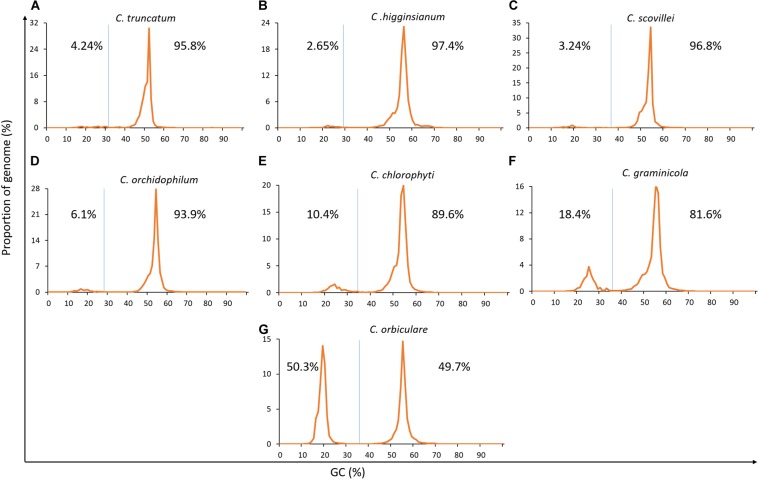
The GC-content plots of seven *Colletotrichum* species arranged from **(A–G)** showing diverse peak heights, shape, and spacing. The GC cut-offs chosen by OcculterCut and used to classify genome segments into distinct AT-rich and GC-equilibrated region types as shown by vertical blue lines. *C. orbiculare*, *C. graminicola* and *C. chlorophyti* had bimodal genomes with strong GC-bias, while rest of the species showed very subtle signatures of bimodality.

The genome-wide analysis of GC-content showed that *C. truncatum* exhibited very subtle signs of bimodal GC-content like three other *Colletotrichum* species, while *C. chlorophyti*, *C. graminicola*, and *C. orbiculare* showed strong bimodality, with the latter showing AT-rich sequences in half of the genome (**Figure 4**). Though the AT-rich region occupied 3.13–50.3% of the genome in all the species, only minor fractions of genes (0–1.2%) were present in these regions in the species where gene annotations are available publically. Nevertheless, all the genomes were analysed for evidence of RIP using RIPCAL. The genome-wide dinucleotide frequency obtained with RIPCAL showed that *C. truncatum* had lower TpA/ApT index (0.82) and a higher (CpA + TpG)/(ApC + GpT) index (1.18) than the threshold expected for RIP affected genomes, viz; 0.89 and 1.03, respectively. Only *C. graminicola* and *C. orbiculare* showed a strong signature of RIP across the genomes based on the RIP indices (**Supplementary Table S2**).

However, alignment-based analysis of the copies of the most prevalent repeat families in *C. truncatum* genome showed signatures of RIP (**Table 5**). The truncated or incomplete elements were discarded and the 14 families with at least 5 intact copies were considered for RIP analysis. Investigation into the dinucleotide bias at sites with C to T transitions showed CpA were the most preferred dinucleotide target sites specific to RIP in MULE-MuDR and Tc-Mariner families of DNA elements and two Gypsy elements, whereas CpT dinucleotides were the preferred target sites in other Gypsy and Copia families. In Gypsy-2 family, both CpA => TpA and CpT => TpT transitions were equally prevalent. The two RIP indices were calculated for all the 14 families. TpA/ApT index ranged from 0.93–1.68 that was higher than the standard value of >0.89 indicative of RIP, while the (CpA + TpG)/(ApC + GpT) index (standard value <1.03) ranged from 0.12–1.21 with families of two DNA elements, MULE-MuDR-1 and Tc1-Mariner-Fot1-1 showing higher values than the threshold expected for RIP (**Table 5**).

**Table 5:** The analysis of RIP indices and dinucleotide bias in TE families.

Repeat family	TpA/ApT index	(CpA + TpG)/ (ApC + GpT) Index	Dinucleotide bias
Copia-1	1.64309	0.123305	CpT
Copia-2	1.655783	0.12236	CpT
Copia-3	1.66795	0.091286	CpT
Gypsy-1	1.622181	0.263649	CpA
Gypsy-2	1.676275	0.144421	CpA and CpT
Gypsy-3	1.589147	0.420066	CpA
Gypsy-4	1.611704	0.119303	CpT
Gypsy-5	1.610562	0.081724	CpT
Gypsy-6	1.589147	0.420066	CpA
MULE-MuDR-1	0.929878	1.055794	CpA
MULE-MuDR-2	1.287078	0.670805	CpA
Tc-Mariner-1	1.136585	1.213974	CpA
Tc-Mariner-2	1.409766	0.748148	CpA


*Colletotrichum higginsianum* was previously reported to have two orthologs of cytosine methyltransferases involved in DNA methylation; viz., *RID* gene (CH63R_07391) responsible for C to T transitions during RIP, and *Dim-2* gene (CH63R_01196), which brings about a potential bias in dinucleotide mutations (Dallery et al., 2017). There were no orthologs of *RID* in *C. truncatum*, but the presence of cytosine-specific methyltransferase domains (PF00145) was detected in two genes, *CTRUNC_007747* and *CTRUNC_010784*, which were homologous to DNA methyltransferase *Dim-2* and DNA repair protein, *RAD8* of other *Colletotrichum* spp., respectively.

### Analysis of SSRs

**FIGURE 5:**
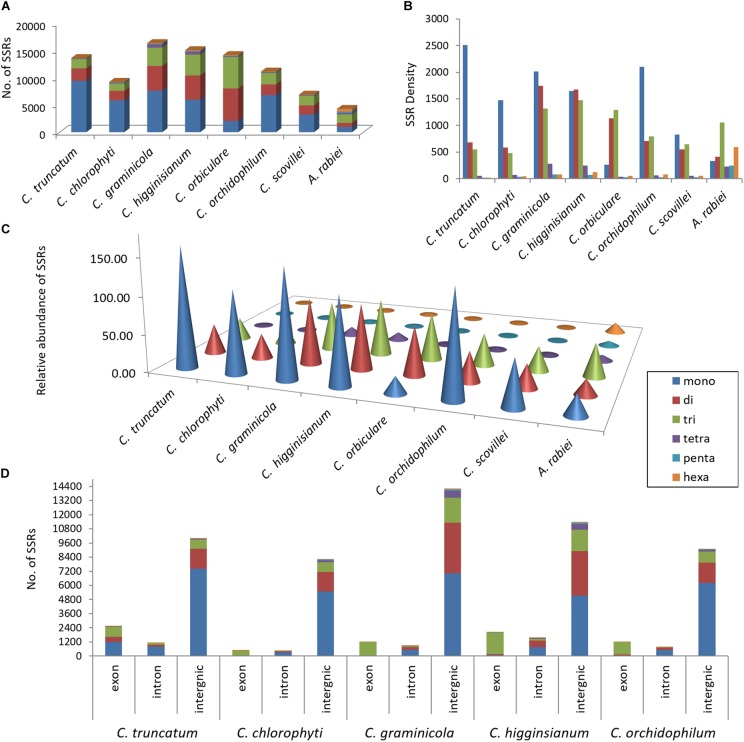
Composition of SSRs in seven *Colletotrichum* species showing the distribution of SSRs in genomes in terms of **(A)** their number, **(B)** relative density (size of each SSR type in bp/Mb of genome), **(C)** relative abundance (number of SSRs/Mb of genome), and **(D)** the number of SSRs in coding (exons) and non-coding regions (introns, intergenic regions). *Ascochyta rabiei* was taken as a reference for accuracy of SSR detection. In all the species, the mononucleotide repeats were the most abundant SSR type except for *C. orbiculare*, in which tri- and di-nucleotide repeats were more predominant. SSRs were mainly concentrated in intergenic regions. Exons had high proportion of trinucleotide repeats in four species, whereas mononucleotide repeats were more than the trinucleotide repeats in coding region of *C. truncatum*.

For more accurate identification of the SSR motifs than those identified by RepeatMasker, genome-wide SSR analysis was performed in *C. truncatum* through a specialised microsatellite identification tool, MISA. A total of 13,597 SSRs were identified in 62 scaffolds including ∼2,000 compound SSRs. The SSRs constituted 0.38% of the entire genome with relative abundance of 234.8 SSRs/Mb and relative density of 3830.7 bp/Mb of genome (**Figures 5A–C**). The mononucleotide repeats (mainly A/T) were the most common SSR types, which represented 69% of all SSRs, while di- and tri-nucleotide repeats represented 17 and 13% of total SSRs, respectively. The nine most abundant motifs (>190), constituting mono-, di- and tri-nucleotide repeats, constituted 93.2% of the total SSRs detected in *C. truncatum* genome. The mononucleotide repeat motifs, A/T and C/G occurred at the highest frequencies of 56.32 and 12.8%, respectively. Other most frequent motifs were dinucleotide motifs, AC/GT (7.17%) and AG/CT (7.12%) followed by a trinucleotide motif, AGC/CTG (2.7%). The majority of tetranucleotide motifs and all the penta- and hexa-nucleotide motifs occurred less than 10 times.

The entire genome was divided into coding region, represented by exons, and non-coding region, represented by introns and intergenic sequences. ∼81% of SSRs concentrated in the non-coding region with more abundance of mono- and di-nucleotide repeats. Tri- and hexa-nucleotide repeats were fairly evenly distributed within exons and intergenic region, while tetra- and penta-nucleotide repeats were concentrated in the latter (**Supplementary Table S3**). Exons had a high proportion of mononucleotide repeats followed by tri- and di-nucleotides. Trinucleotide repeats in the coding region have the potential to alter protein structure and function. Analysis of all the trinucleotide motifs encoding amino acids in exons showed the highest frequency of motifs coding for alanine (102) followed by serine (84) and arginine (81). Other highly frequent repetitive amino acids (>60) translated by trinucleotide repeats were lysine, leucine, glycine, glutamine, proline and cysteine.

**FIGURE 6:**
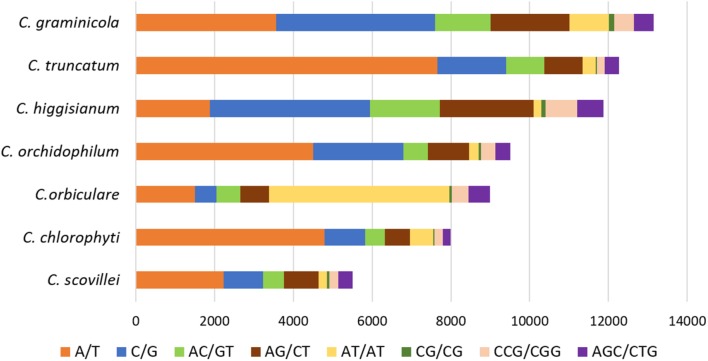
The common repeat motifs occurring at high frequencies in the genomes of seven *Colletotrichum* species analysed. The mononucleotide repeats A/T and C/G were the most frequent motifs in all the species, except *C. orbiculare* which showed the most frequent dinucleotide motif AT.

The comparative analysis of SSRs among seven *Colletotrichum* species revealed the largest SSR component in *C. higginsianum* and the smallest in *C. scovillei* with 16,342 and 6,882 SSRs, respectively (**Figure 5A**). Mononucleotide repeat motifs were the most common type of SSRs in all the species, except in *C. orbiculare* which had the largest proportion of tri- and di-nucleotide repeats. However, the total number, relative density and relative abundance of SSRs in *C. orbiculare* were comparable to other species despite its largest genome size of ∼91 Mb (**Figures 5A–C**). The comparative analysis of the most frequent motifs of each SSR type was carried out among the seven *Colletotrichum* species. The common motifs occurring at high frequencies in all the species belonged to mono-, di- and tri-nucleotide repeats and showed a strikingly different pattern of distribution in different species (**Figure 6** and **Supplementary Table S4**). The tetra-, penta-, and hexa-nucleotide repeat motifs were less frequent than the first three types (**Supplementary Table S4**). A/T was the most abundant motif in *C. scovillei*, *C. orchidophilum*, and *C. chlorophyti*, followed by C/G, similar to the pattern shown in *C. truncatum*. The third most frequent motif in these species was AG/CT. In *C. graminicola* and *C. higginsianum*, C/G motifs were more abundant than A/T, followed by AG/CT and AC/GT. Interestingly *C. orbiculare* showed a different trend than rest of the species in which a dinucleotide AT was the most predominant motif followed by a trinucleotide motif AAT/ATT. Other frequent motifs in this species included A/T, AG/CT, and AC/GT.

**FIGURE 7:**
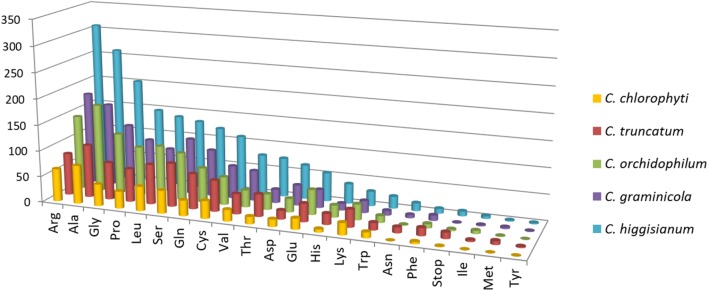
The abundance of each amino acid encoded by trinucleotide repeat motifs in exons of five *Colletotrichum* species. The most abundant motifs encoded alanine or arginine in all the species. Ala, alanine; Arg, arginine; Asn, asparagine; Asp, aspartate; Cys, cysteine; Gln, glutamine; Glu, glutamate; Gly, glycine; His, histidine; Ile, isoleucine; Leu, leucine; Lys, lysine; Met, methionine; Phe, phenylalanine; Pro, proline; Ser, serine; Thr, threonine; Trp, tryptophan; Tyr, tyrosine; Val, valine.

The exonic, intronic and intergenic SSRs were analysed in all the species, except *C. orbiculare* and *C. scovillei* due to the unavailability of gene annotations in.gff format. It was observed that the largest proportion of the total SSRs was concentrated in intergenic regions, followed by exons with slightly higher concentration than that in the introns (**Figure 5D**). Only in *C. truncatum* exons, SSR concentration was more than double than that in introns (**Supplementary Table S3**). Trinucleotide repeats were predominant in the exonic regions in all species, except for *C. truncatum* in which mononucleotide repeats formed the largest fraction, followed by tri- and di-nucleotide repeats (**Figure 5D** and **Supplementary Table S3**). Tetra-, penta-, and hexa-nucleotide repeats were mostly concentrated in the intergenic regions in all the species. The analysis of trinucleotide repeat motifs revealed different capacities of coding for amino acids in proteins of different species with the highest number of motifs in *C. higginsianum* followed by *C. graminicola*, *C. orchidophilum*, *C. truncatum*, and *C. chlorophyti* (**Figure 7**). The most frequent motifs in all the species were coding for alanine, arginine, glycine, leucine, and serine. In *C. graminicola* and *C. higginsianum*, the repeats coding for arginine were the most abundant motifs, followed by alanine and glycine, while in *C. orchidophilum* and *C. chlorophyti*, alanine and arginine were coded most frequently. In *C. truncatum*, the most frequent repeats were coding for alanine and serine. Serine was among the most frequently coded amino acids in all the species, while the least frequent repetitive amino acids were tyrosine, methionine and isoleucine that were totally absent in *C. chlorophyti*.

## Discussion

Fungal phytopathogens with broad host range adapt to changing environments through genetic variations caused by a variety of mechanisms. Meiotic recombination is the most common phenomenon in sexually propagating species that results in different novel alleles, whereas in asexual species, genomic rearrangements foster adaptation (Seidl and Thomma, 2014). The genomes of a number of fungal plant pathogens that have been sequenced so far, provide an important resource to study their genome architecture. High levels of genomic plasticity and variations in genome architectures were observed even between related species of pathogenic fungi, mainly due to the differences in TE content (Möller and Stukenbrock, 2017). Activity and spread of TEs can alter the genome architecture substantially, resulting in the expansion of certain gene families and in rapid evolution of virulence determinants, notably effectors (Castanera et al., 2016). In fungi, the TEs usually constitute 1–25% of the genome, which is much less than that found in animals and plants, which have upto 60–90% of their genomes composed of TEs (Castanera et al., 2017a). The assembly of repeats with whole genome sequencing data is difficult since the redundant reads corresponding to the repetitive sequences collapse by the use of assembly algorithms. Recent advancements in the long read sequencing technologies have enabled the assembly across repetitive regions by spanning the repeats in single reads which may contain an entire TE sequence. These long reads help in obtaining contiguous genome assemblies, which facilitate analysis of TEs, SSRs and other repeats to assess their impact on genome evolution.

In this study, we resequenced the genome of chilli anthracnose fungus, *C. truncatum* through PacBio SMRT long read sequencing technology in order to improve the existing high-quality draft assembly by filling the gaps and to get a clear picture of the repetitive element landscape. Hybrid scaffolding resulted in a refined genome which was more compact than the previous assembly with significant reduction in number and size of gaps, though only a marginal reduction in the total number of scaffolds was observed. This may be due to the smaller reads of inserts obtained and low sequence coverage achieved by the three SMRT cells used in this study. The size selection of longer reads (∼20 kb or more) and sequencing in more SMRT cells may provide sufficient sequence data to reduce the number of scaffolds to a great extent. However, the reduction of gaps in the current refined assembly was substantial enough to carry out the analysis of repetitive elements. The repeat content in the previous genome assembly (1.2%), which was under-estimated owing to the unassembled repeat-rich regions, had improved to ∼6.8% in the refined assembly. The TE content, as estimated using a combined library of *de novo* repeats in genome and repeats in Repbase database, was comparable to the TE content previously reported in fungi (Castanera et al., 2017a).

The less fragmented genomes of six other *Colletotrichum* species were also subjected to comparative analysis of TEs. The genome size of all the species ranged from ∼48–58 Mb, except for *C. orbiculare*, which had a large genome of ∼91 Mb. TE fractions were consistent with the previous reports in *C. graminicola* (O’Connell et al., 2012) and *C. higginsianum* (Dallery et al., 2017) but not in *C. orbiculare* which had 44.9% of TEs, ∼5 times higher than a previous report (8.3%) that showed enrichment of DNA elements followed by LTRs in its genome (Gan et al., 2013). Contrastingly, in our study, TE landscape of *C. orbiculare* was found to have high proportion of Copia elements (12%) and a small fraction of DNA elements (0.02%). Unknown elements formed the highest proportion of total TEs (26.4%), which was the case for all the genomes analysed. This indicates that most repetitive sequences in *C. orbiculare* as well as in other *Colletotrichum* species are unique, as they were not found in other fungal genomes in Repbase database. The discovery of such large number of species-specific TEs in all the genomes and Copia elements in *C. orbiculare* could be due to the addition of the repeat libraries derived from each genome using *de novo* repeat-finding approach to the updated Repbase database (containing 2460 fungal repeats) used in our analysis. A detailed analysis of these TEs would identify and distinguish the complete and partial elements with active protein-coding sequences from the relics of TEs. However, high LTR content of *C. orbiculare* suggests that the genome size expansion could be attributed to the past transposition events of LTRs in this species.

*Colletotrichum higginsianum* genome had the largest proportion of LTRs, represented mainly by Gypsy and Copia elements, followed by TC1-mariner superfamily of DNA elements of TIR order, an observation similar to that reported in a previous study (Dallery et al., 2017). Gypsy and Tc1-mariner Fot1 elements were the most abundant TEs in other *Colletotrichum* species as well, except for *C. orbiculare*. These observations could be an underestimation due to the missing TEs with long and repetitive sequences in most of the assemblies which had multiple scaffolds, including *C. truncatum*. Analysis of unassembled reads in such species might uncover many TEs which are not detected in the assembled genomes, as observed in case of *Amanita* fungi (Hess et al., 2014). Nevertheless, the combination of *de novo* and homology-based detection of repeats identified most of the TEs in assembled genomes of seven *Colletotrichum* species, thus laying a strong base for the future studies. Our findings were consistent with the previous reports from other fungi in which LTRs formed the major fraction of TE content (Muszewska et al., 2011), and DNA elements were found to be less expanded in pathogenic fungi than in non-pathogenic taxa (Elliott and Gregory, 2015; Castanera et al., 2017a). Gypsy elements are known to be the most successful TEs in fungi capable of autonomous transposition to increase their copy numbers in the genomes (Elliott and Gregory, 2015), and *Colletotrichum* fungi were no exception. The largest genome size among *Colletotrichum* species sequenced so far is that of *C. orbiculare* and the role of Copia elements in its genome size expansion is evident from our study.

The number of intact LTRs varied widely in all the species analysed, which corresponded to the fraction of the total genome occupied by these elements. *C. truncatum* had 562 intact elements, 1.8–5 times higher than the number of LTRs that were detected in other species, except *C. orbiculare* that had 845 elements, the highest among all the species analysed. The estimation of the age of insertion was based on the sequence divergence between the 5′ and 3′ LTRs of each LTR retrotransposon that were assumed to be identical at the time of insertion. The three *Colletotrichum* species with less fragmented genome showed a recent amplification burst of the LTR retrotransposons inserted between 0–10 MYA. In *C. truncatum*, 58% of the LTRs were amplified in last 3 MYA, while 30% were amplified in 3–10 MYA. 78 LTR pairs were identical in this species. In *C. higginsianum* and *C. scovillei*, 40–48% LTRs were amplified in last 10 MYA, while 35 and 24 identical LTR pairs were observed in these species, respectively. This suggests that most of the LTRs in these species are recent and putatively active elements that had not accumulated evolutionary mutations. The recent amplification bursts of LTRs might indicate that these species are currently undergoing a period of genome expansion. The continued expansion of these retrotransposons might play a role in shaping the genome architecture in these fungi. Similar observations were made in some members in the order Boletales of brown-rot Basidiomycetes, viz., *Coniophora* species and *Serpula lacrymans* (Castanera et al., 2017b).

In the other four species, the profile of LTR insertion age was completely different, with more ancient bursts of amplifications as compared to only a few recent LTR insertions from 0–10 MYA. The presence of intact and ancient LTRs show that these elements retained the conserved domains needed for transposition and might have contributed to their diversification. The high proportion of recently amplified LTRs in *C. higginsianum*, *C. scovillei* and *C. truncatum* may be more reliable due to their nearly gapless or highly contiguous assemblies, leading to the identification of more complete LTR profiles. Relatively more fragmented genome assemblies of the other four species might have led to an underestimation in the number of young LTRs due to the presence of highly repetitive and identical sequences in them as observed in *Pisolithus tinctorius* and *Hydnomerulius pinastri* (Castanera et al., 2017b). The completion of genome assemblies of such fungi might therefore be more challenging with conventional sequencing approaches.

There is an increasing evidence for the role played by TEs in establishing genomic plasticity during host–microbe interactions (Raffaele and Kamoun, 2012; Dong et al., 2015). TEs have been shown to mediate genome variability in plant–fungal interactions, and drive their coevolution. In fungal genomes, TEs are often clustered to form AT-rich islands or TE-rich and gene-sparse regions within the core chromosomes, or reside on the accessory chromosomes with most of the effector genes in close proximity to these regions. The ‘two-speed’ genome hypothesis was proposed based on these observations which states that many pathogenic fungi have gene-poor and TE-rich genomic compartments with higher rates of mutations and hence evolve more rapidly than rest of the genome (Faino et al., 2016). The genome structure and function is considerably affected by TE activity, which may result in gene disruption, genomic rearrangements like translocation, duplication or deletion of genomic regions or may affect the expression of proximal genes like effectors. This leads to effector diversification and formation of novel effectors that helps the pathogens to adapt to different hosts and dynamic environmental conditions (Dong et al., 2015; Faino et al., 2016).

Since excessive TE propagation may have deleterious effects on the organism, their activity is generally regulated by genome defence mechanisms such as RIP, DNA methylation and RNAi (Muszewska et al., 2011). RIP is a common genome defence mechanism in fungi with sexual lifestyle that prevents sequence duplication by G:C to A:T transition mutations in and around TEs in pre-meiotic stages (Cambareri et al., 1989; Hane and Oliver, 2008). In general, two-speed genomes of many pathogenic fungi show GC-bias in genomes, where AT-rich regions represent a strong signature of RIP (Dong et al., 2015; Faino et al., 2016). DNA methylation is an epigenetic mechanism that increases methylated cytosines in and around genes and transposons. The studies involving genome-wide methylation analyses in fungi have shown that methylation of cytosines in CpG context controls TE proliferation (Zemach et al., 2010). RNAi pathway genes are triggered for meiotic silencing of TEs if aberrant RNAs are detected (Fulci and Macino, 2007).

*In silico* methods to detect AT-rich islands and RIP showed that there was bimodality but no distinct GC-bias or compartmentalisation into TE-rich regions in the genomes of *C. truncatum*, *C. higginsianum*, *C. scovillei*, and *C. orchidophilum*, whereas *C. chlorophyti*, *C. graminicola*, and *C. orbiculare* clearly showed a strong GC-bias (**Figure 4**). Moreover, AT-rich regions were also gene-sparse in the genomes of all the species where gene annotations are publically available. Interestingly, *C. orbiculare* had more of distinct AT-rich region (50.3%) than GC-equilibrated region, while *C. graminicola* had 18.4% of AT-rich region. These figures were consistent with the previous reports and RIP indices in these two species (discussed below) suggest that RIP could have led to the two-speed genomes in these fungi (Gan et al., 2013; Testa et al., 2016). Rest of the species, including *C. truncatum*, showed very subtle signatures of GC-bias, suggesting that the GC-content is almost consistent throughout the genome. Some fungi like *Blumeria* spp. and *Puccinia* spp. have a high repeat content but unimodal GC-content distributions with no compartmentalisation into AT-rich and GC-equilibrated regions (Testa et al., 2016). This indicates that the *Colletotrichum* genomes with weak RIP indices either exhibit no or limited RIP or have a uniform RIP throughout the genome, and do not harbour distinct AT-rich, RIP-affected regions, as exemplified by *C. higginsianum* (Dallery et al., 2017).

The two RIP indices, (CpA + TpG)/(ApC + GpT) and TpA/ApT, were calculated based on the dinucleotide frequencies across genome. TpA/ApT ≥ 0.89 and (CpA + TpG)/(ApC + GpT) ≤ 1.03 indicate a high probability of occurrence of RIP in the genomes (Hane and Oliver, 2008). In *C. truncatum*, a low TpA/ApT index (0.81) and a high (CpA + TpG)/(ApC + GpT) index (1.17) implied a weak RIP, whereas in *C. graminicola* and *C. orbiculare*, the values of these indices were indicative of strong RIP activity (**Supplementary Table S2**). In *C. graminicola*, owing to the sexual mating stages (Chen et al., 2002), it is likely that RIP could be active in its genome. In *C. orbiculare* RIP activity was observed in a previous study also, which explained the expansion of AT-rich sequences in its genome (Gan et al., 2013). In *C. higginsianum*, the RIP indices implied a weak RIP in the present study, while an earlier study suggested that some TE families were RIP-affected in this fungus, which has not been reported to have sexual stages so far (Dallery et al., 2017). In the same study, the homolog of RIP defective gene (*RID*), a gene known to be essential for RIP (Freitag et al., 2002), was found in the genome of *C. higginsianum*. The evidence for RIP mutations was found previously in other asexual species like *C. cereale* (Crouch et al., 2008). In some fungi like *Purpureocillium lilacinum*, in which the sexual stages are not verified yet, RIP indices indicated weak RIP, but retention of *RID* gene indicated a role of RIP at some developmental stages during its evolution (Xie et al., 2016). In entomopathogenic *Metarhizium* species, during transformation from very narrow host range specialists to a wide range generalists, the expansion of gene families and loss of sexuality were associated with lower RIP activity (Hu et al., 2014).

In order to find evidence for RIP activity in *C. truncatum*, some of the most prevalent TE families were analysed. The copies in each family were aligned with the longest sequence with highest GC content taken as reference. The copies with >400 bp size and >80% identity were retained since these are the prerequisite conditions for RIP to act. Only those families with >5 copies were considered for analysis. All the 14 families of LTR and DNA elements, except two, showed strong signatures of RIP as evident from the two RIP indices. Different fungal species show different bias toward certain dinucleotides that are preferential RIP targets (Amselem et al., 2015). CpA or CpT dinucleotide bias was observed in all the families in *C. truncatum*, except Gypsy-2 in which both of the above dinucleotides were preferred equally. CpA is the preferred dinucleotide target site for RIP in most fungi like *Neurospora*, *Manaporthe* species, though CpG and CpT may also be the RIP sites in some fungi like *Aspergillus* species (Amselem et al., 2015). RIP requires the *RID* gene that encodes a C5-DNA-Methyltransferase (MTase) of the Dnmt1 family. *RID*-mediated deamination of methylated cytosine and its subsequent replacement with thymine is thought to occur during RIP (Amselem et al., 2015). However, no homologs of *RID* were found in the genome of *C. truncatum* and no sexual stage has been detected in this species yet. Though the signatures of RIP found in some of the TE families suggest that RIP might have been an active mechanism of TE silencing during ancestral sexual stages of *Colletotrichum* fungi or that natural meiosis occurs cryptically but is difficult to detect in the laboratory (Dallery et al., 2017).

Repeat-induced point is known to be an active TE silencing mechanism in several fungi ranging from *Neurospora crassa* (Galagan et al., 2003) to *Mycosphaerella* spp. (Santana et al., 2012; Dhillon et al., 2014). In *Cochliobolus heterostrophus*, RIP was found to be selective for TEs near the coding regions (Santana et al., 2014). Since RIP has been proposed to prevent gene duplications, a reduction in the numbers of gene families is observed in genomes affected with RIP (Galagan et al., 2003). RIP should be inactive for duplicated genes to function. In general, the gene families like peptidases, kinases, transporters, carbohydrate active enzymes are highly expanded in *Colletotrichum* species and more so in *C. truncatum* (Rao and Nandineni, 2017), which might be a likely consequence of the lower levels of RIP in its genome.

To test whether the important gene categories, including effectors, were associated with repetitive elements in *C. truncatum*, distances of these gene sets from the repetitive sequences were compared with the distances of the randomised control sets of genes of same size as those of each category. There was no significant association of secretory genes, effectors or genes within the secondary metabolite clusters with repeat elements. In a previous study, a statistically significant association was found for certain TE families with the effector genes and SM cluster genes in *C. higginsianum* (*p* < 0.001) than with random sets of genes sampled with 10,000 permutations (Dallery et al., 2017). Hence, the permutation test with higher number of random gene samples was considered to be more appropriate for such analyses. It was used to analyse the mean distance between TEs and the functional categories of genes as well as random sets of genes taken from whole genome in *C. truncatum*. This test used 10,000 permutations of random gene samples which showed a significant association between TEs and secretome (*p* = 0.0001), effectors (*p* < 0.001) and genes in secondary metabolism gene clusters (*p* < 0.05).

In *Leptosphaeria maculans*, secreted proteins, including effectors, were found to be significantly closer to TEs than random genes, and were subjected to extensive RIP (Rouxel et al., 2011). Similar inferences were made in other fungi like *Blumeria graminis* and *Phytophthora infestans*, in which both secreted and effector-like genes were closely associated with repetitive elements (Derbyshire et al., 2017). Although these fungi do not exhibit active RIP, they do show compartmentalisation of effector genes in TE-rich regions (Haas et al., 2009; Pedersen et al., 2012; Derbyshire et al., 2017). In another fungus with broad host range, *Sclerotinia sclerotiorum*, no significant differences among these categories were observed and the lack of bimodality despite the existence of RIP in its genome could indicate absence of specific RIP-affected genomic regions such as those observed in *L. maculans* (Rouxel et al., 2011; Derbyshire et al., 2017). In the family *Magnaporthaceae*, there was no evidence of two-speed genome evolution and the proximity of genes to repetitive elements had no influence on diversification of effectors in some of the fungi belonging to this family (Okagaki et al., 2016). These observations suggest that TE activity may contribute in effector or secreted protein evolution in fungi to a certain extent and lack of GC bias and genome compartmentalisation into gene-poor, AT-rich and TE-rich regions is not a strong evidence but an indication of lack of RIP or weak RIP. Though *C. truncatum* like *C. higginsianum*, lacked repeat-rich islands, proximity of TEs with the secreted proteins, effectors and genes in secondary metabolite clusters suggested that these gene categories might be subjected to rapid diversification with each transposition event and thus contribute to the pathogenicity, virulence and broad host-range of these *Colletotrichum* species.

Though *C. truncatum* genome did not show the homologs of genes required for RIP activity, the presence of genes with cytosine methyltransferase domain indicates that DNA methylation might regulate the activity and spread of TEs in its genome (Amselem et al., 2015). In several fungal pathogens, TE-rich regions are present in highly condensed heterochromatin, which is directed by DNA methylation in epigenetic regulation (Seidl et al., 2016) and can influence the expression of TEs as well as the genes in their vicinity (Soyer et al., 2015). This epigenetic defence mechanism is active in some fungi to control their expression and proliferation (Zemach et al., 2010; Castanera et al., 2016). The active Class I TEs, which form a major fraction of repeats in fungi, can be detected through expression analysis since these elements show high transcriptional levels (Castanera et al., 2016). There is scope for further studies like genome-wide methylation analyses and TE or gene expression analyses to look for evidence of active DNA methylation as silencing mechanism to control the TE activity in *C. truncatum* and other *Colletotrichum* species.

Apart from TEs, SSRs or microsatellites are the other major repeat types in fungal genomes that shape the genome architecture and are often used as molecular markers in population genetics studies (Karaoglu et al., 2005). The SSRs in the genome of an organism evolve through replication slippage, point mutation, and recombination, which generate novel genetic loci, eventually leading to the genetic diversity (Li et al., 2004). The strains of *C. truncatum* and other *Colletotrichum* species were differentiated using ISSR (Ratanacherdchai et al., 2010; Mahmodi et al., 2014; Saxena et al., 2014), RAPD (Chen et al., 2002; Saxena et al., 2014), and microsatellite markers (Ranathunge et al., 2009; Rampersad, 2013; Sharma et al., 2014; Diao et al., 2015). 27 microsatellite markers were used to estimate the diversity of 52 isolates of *C. truncatum* from India, Sri Lanka, and Thailand (Ranathunge et al., 2009). Genetic diversity estimation based on SSRs showed high diversity among *C. truncatum* isolates from India (Sharma et al., 2014), while evidence of sexual recombination, and geographic differentiation were obtained for *C. truncatum* isolates in China (Diao et al., 2015). There are major limitations of other molecular markers and the conventional methods associated with isolation of SSRs in the whole genome. Construction of SSR-enriched libraries or screening of small insert genomic DNA libraries is a time, cost and labour intensive exercise. Traditional methods also have issues with reproducibility and are often not sufficient for evaluation of strain variations (Mahfooz et al., 2017). With increasing number of fungal genomes being sequenced through NGS and development of a number of *in silico* tools, genome-wide analysis of SSRs has become much simpler and quicker. In our study, the refined genome of *C. truncatum* was mined for SSRs and their distribution and abundance was determined in different genomic regions. Genome-wide comparative analyses of SSR distribution were carried out among *C. truncatum* and six other species. This is the first report of genome-wide SSR detection for any *Colletotrichum* species based on whole genome sequencing, to the best of our knowledge, which could be useful to develop new microsatellite markers for studies on population genomics, genetic diversity and evolution.

Over 13,500 microsatellites were identified in the *C. truncatum* genome and their abundance and distribution in entire genome, as well as in different coding and non-coding regions was compared with *Colletotrichum* species for which annotation files (.gff3) were available. Genome-wide analysis of all seven *Colletotrichum* species showed that *C. higginsianum* and *C. graminicola* had the highest SSR densities (>5200 bp/Mb of genome), followed by *C. truncatum* (3831 bp/Mb of genome). A detailed analysis revealed that ∼70% of SSR-content in *C. truncatum* was composed of mononucleotide repeats (mainly A/T repeats). In rest of the species too, mononucleotide repeats formed the largest SSR type, except for *C. orbiculare*, in which the tri- and di-nucleotide repeats covered the major proportion of the genome. SSR-content did not correspond to the genome size expansion in *C. orbiculare*, which showed lower SSR density than four other *Colletotrichum* species. Our findings were consistent with the notion that fungi have much lesser and shorter SSRs than other eukaryotes and there is no correlation between their genome size expansion with SSR density (Tóth et al., 2000; Lim et al., 2004; Karaoglu et al., 2005). This further supports the argument that the role of TEs, especially LTRs, is more significant in fungal genome expansion than SSRs, as seen in other organisms with large genomes in plant and animal kingdoms.

The analysis of most frequent repeat motifs revealed that phylogenetically closer species had similar frequencies of the most abundant repeats. In *C. graminicola* and *C. higginsianum*, which belong to the sister clades of graminicola and destructivum species complexes, C/G represented the most abundant motif, while the most frequent motif was A/T in *C. truncatum* belonging to truncatum clade, *C. scovillei* of acutatum clade, and the two singleton species, *C. orchidophilum* that clusters basal to the acutatum clade and *C. chlorophyti*, both of which do not belong to any of the species complexes identified in the genus *Colletotrichum* to date (Jayawardena et al., 2016). *C. orbiculare* was the only species belonging to a separate orbiculare clade with a dinucleotide AT and trinucleotide AAT/ATT representing the most frequent motifs. However, all the species had AG/CT and AC/GT among the top five motifs with high abundance. The inclusion of more species with less fragmented genomes may give a greater insight into the SSR composition in phylogenetically closer species, enabling study of evolutionary aspects of their proliferation and development of SSR markers for species identification. Nevertheless, designing primers based on the identified loci harbouring longest SSRs, which display high polymorphism, would be useful as molecular markers for identification and characterisation of different *Colletotrichum* species and strains.

The distribution of SSRs in exon, intron and intergenic regions was similar for all other species with intergenic regions harbouring 73–90% of SSRs, dominated by mononucleotide repeats. Trinucleotide repeats were highly predominant in the exonic regions in all species except for *C. truncatum*, in which exons had more mononucleotide repeats than tri- and di-nucleotide repeats. (**Figure 5D** and **Supplementary Table S3**). Tetra-, penta-, and hexa-nucleotide repeats were mostly concentrated in the intergenic regions in all the species. There was substantial evidence for non-random distribution of SSRs across protein-coding regions, UTRs, and introns (Li et al., 2004; Vieira et al., 2016). Similar observations were made in other fungi in which trinucleotide repeats were selected as opposed to other types of SSRs in open reading frames and 5′ upstream regions, like in edible mushrooms like *Pleurotus ostreatus* (Qu et al., 2016), forest pathogen *Heterobasidion irregulare* (Gonthier et al., 2015), *Aspergillus* species (Mahfooz et al., 2017) and the mycoparasitic *Trichoderma* species (Mahfooz et al., 2016), *Agaricus bisporus* (Foulongne-Oriol et al., 2013); and in yeast (Richard and Dujon, 1996), which resembles humans in terms of distribution and stability of trinucleotide repeats (Subramanian et al., 2003). Since SSRs within genes evolve through mutational processes, they have the potential to generate novel alleles at the loci harbouring them. SSR expansions or contractions in the coding sequences may alter gene products via frameshift mutations and/or regulate gene expression and transcription that eventually lead to phenotypic changes and genetic diversity. Hence, SSRs within genes are subjected to a strong selective pressure and have better tolerance for frame shift mutations in coding regions due to high prevalence of repetitive trinucleotide motifs, which may alter the protein structure and function by altering the number of specific translated amino acids (Li et al., 2004; Katti et al., 2008). The high prevalence of mono-and di-nucleotide motifs in exons of *C. truncatum* might indicate a higher propensity of formation of novel proteins that warrant further investigations of the genes disrupted by these SSRs in the future.

The analysis of trinucleotide motifs in all the species revealed different capacities of coding for amino acids in proteins of different species, but the most abundant amino acids in all the species were either small (alanine, glycine, proline) or hydrophilic (arginine). Similar trend was observed in other fungi like *P. ostreatus* (Qu et al., 2016). The motifs encoding small/hydrophilic amino acids were reported to be better tolerated in many proteins, which may ensure their survival in a population (Katti et al., 2008). The pattern of trinucleotide motifs in exons encoding amino acid repeats was similar in phylogenetically closer species, a trend observed in the analysis of most frequent repeat motifs in the genomic context as well. *C. higginsianum* and *C. chlorophyti* had the highest and lowest numbers of repetitive amino acids, respectively. The most frequent motifs in *C. graminicola* and *C. higginsianum* coded for arginine, alanine, and glycine, while in *C. truncatum*, repeats encoding alanine and serine were the most abundant ones. In *C. orchidophilum* and *C. chlorophyti*, alanine was encoded more frequently than arginine. Serine-rich sequences are reported to be associated with membrane transporter proteins (Mar-Alba et al., 1999), and in our study we found it was among the most frequently coded repetitive amino acids in all the species. In-depth analysis of amino acid changes due to SSRs in coding region would be interesting to study the implications of changes in the amino acid sequence, structure and function of the proteins which are disrupted by SSRs.

## Conclusion

We have generated a refined genome sequence of an important broad host range phytopathogenic fungus, *C. truncatum*. We explored the genome architecture of six other *Colletotrichum* spp. along with *C. truncatum* by examining the repetitive element landscape, mainly TEs and SSRs. Retrotransposons, mainly Gypsy and/or Copia elements formed the largest fraction of TEs in all species. The estimation of insertion time of full-length LTRs showed recent bursts of LTR amplifications in *C. truncatum*, *C. higginsianum* and *C. scovillei* while ancient bursts of amplifications in *C. graminicola*, *C. orbiculare*, *C. chlorophyte* and *C. orchidaceae*. The absence of GC-bias or repeat-rich regions in *C. truncatum* contrasted the two-speed genome hypothesis proposed for many of the filamentous fungi and oomycetes. However, the proximity of TEs with secretory genes, effectors or genes within SM clusters was significantly high as compared to the random genes. The most prevalent TE families showed signatures of RIP, but absence of homologs of genes required for RIP and lack of sexual stages suggests ancestral activity of RIP machinery. Though there was no direct evidence for the DNA methylation in the TEs, the presence of genes like cytosine methyltransferase suggested that this could be the active TE silencing mechanism in *C. truncatum*. SSRs formed a small fraction of total genome and were mainly concentrated in intergenic regions. Comparative analysis of SSRs in *Colletotrichum* species suggested that certain specific repeat motifs in the genome and trinucleotide motifs in exons had a similar distribution in phylogenetically closer species. This study holds great potential for genetic diversity and evolutionary studies based on repeat families and microsatellite-based molecular markers in the future.

## Data Availability

The datasets generated for this study can be found in the figshare repository and can be accessed using the following links:

PacBio filtered subreads: https://figshare.com/s/55d00270ea0b82ab08e1.

*Colletotrichum truncatum* refined assembly, annotations and repeat analysis: https://figshare.com/s/22f3b4857cd5b79562ba.

## Author Contributions

SR and MN conceived and designed the experiments. SR carried out the DNA extraction, library preparation, PacBio sequencing and assembly. SR, SS, and VO carried out the repeat analysis. SR and MN wrote the paper. All authors read and approved the final manuscript.

## Conflict of Interest Statement

The authors declare that the research was conducted in the absence of any commercial or financial relationships that could be construed as a potential conflict of interest.

## Footnotes

1 https://sourceforge.net/projects/pb-jelly/

2 http://www.repeatmasker.org/RepeatModeler.html

3 http://www.repeatmasker.org/

4 https://www.R-project.org

5 http://pgrc.ipk-gatersleben.de/misa/misa.html
